# Thin bronchoscope-parallel, endotracheal tube-guided stent deployment for cervical tracheoesophageal fistula after anti-cancer therapy: a case report and literature review

**DOI:** 10.3389/fmed.2026.1816744

**Published:** 2026-05-07

**Authors:** Xin-Di Zhang, Yang Bai

**Affiliations:** 1 Department of Pulmonary Disease (Department of Respiratory and Critical Care Medicine), Gansu Provincial Hospital of Traditional Chinese Medicine, Lanzhou, China; 2 Department of Respiratory and Critical Care Medicine, The First Affiliated Hospital of Chongqing Medical University, Chongqing, China

**Keywords:** endotracheal tube, malignant tracheoesophageal fistula, postgraduate education, stent deployment, thin bronchoscope

## Abstract

**Background:**

Malignant tracheoesophageal fistula (mTEF) is a life-threatening complication of esophageal and lung cancers, often presenting significant management challenges, especially in post-radiotherapy patients with anatomical airway distortion. This case report describes a feasible modified approach for closing a cervical mTEF with anatomical airway distortion that precludes laryngeal mask airway (LMA) placement or rigid bronchoscopy. This approach utilizes thin bronchoscope-parallel, endotracheal tube (ETT)-guided stent deployment, without the need for fluoroscopy.

**Case presentation:**

A 79-year-old male with a 5-year history of stage IIIB (T3N1M0) thoracic esophageal squamous carcinoma (post-definitive chemoradiation and pembrolizumab immunotherapy with complete response) presented with 20 days of swallowing-induced immediate cough (Ono’s sign), purulent yellow sputum (30 mL/day), exertional dyspnea (New York Heart Association Class II), and subsequent fever. Diagnostic workup confirmed a cervical mTEF 5 cm below the glottis, with post-radiation airway stiffness precluding LMA placement or rigid bronchoscopy. Under general anesthesia, a 7.5-mm cuffed ETT was positioned 1 cm above the fistula under direct bronchoscopic visualization with real-time distance measurement, and a 3.8-mm thin bronchoscope was inserted in parallel to provide real-time visualization for the stent deployment. An 18-Fr delivery system with a 20 mm (diameter) × 40 mm (length) partially covered Ultraflex metallic stent was deployed via the ETT, achieving immediate and complete mTEF closure. Anesthesia was maintained with propofol-remifentanil infusion and volume-controlled ventilation (tidal volume 6 mL/kg, respiratory rate 16/min, fraction of inspired oxygen 50–60%) with continuous oxygen saturation and end-tidal carbon dioxide monitoring. The patient was extubated promptly, transitioned to oral feeding, and remained aspiration-free for 3 months with no complications. He developed recurrent coughing at 3 months post-procedure and died after declining further treatment.

**Conclusion:**

This single-stage, fluoroscopy-free ETT-parallel thin bronchoscope approach demonstrates preliminary feasibility and short-term safety for high cervical mTEF in post-radiotherapy patients with anatomical airway distortion where LMA or rigid bronchoscopy is unfeasible. Individualized airway management, precise stent selection, and multidisciplinary collaboration are critical for optimizing symptom control, and this approach might serve as a practical short-term palliative alternative in resource-limited settings. These findings are hypothesis-generating and require validation in larger studies.

## 1 Introduction

Malignant tracheoesophageal fistula (mTEF) is a life-threatening complication characterized by a pathological communication between the trachea and esophagus, occurring in 5–15% of esophageal carcinomas and less than 1% of primary lung cancers (1, 2). It typically arises from direct tumor invasion or necrosis induced by anti-cancer therapy, resulting in classic coughing while swallowing (Ono’s sign) (3), severe aspiration pneumonia, malnutrition, and a median survival of 6–12 weeks (1, 2). Given the poor prognosis and short expected survival time, minimally invasive endoscopic stenting has emerged as the primary palliative treatment, aiming to close the fistula, prevent aspiration, and improve quality of life (QoL) (2, 4–6). Single esophageal stenting with a covered self-expandable metallic stent is the optimal choice for middle- and lower-thoracic esophageal mTEF without airway stenosis, while single airway stenting is preferred for the cervical (upper tracheal) mTEF where esophageal stenting is technically challenging or pre-existing tracheal stenosis exists (7). Double esophageal and airway stenting can be used when fistula closure is not achieved with esophageal or airway prosthesis deployment alone (8).

Conventional interventional approaches for single airway stenting, such as rigid bronchoscopy with jet ventilation or fluoroscopy-guided stent insertion, are often impractical for frail patients or unavailable in resource-limited medical settings (9). In patients with cervical mTEF, laryngeal mask airway (LMA) with mechanical ventilation may serve as an alternative for airway stenting (10). However, the management of cervical mTEF in post-radiation patients presents unique technical challenges. The direct tumor invasion and radiation-induced fibrosis can lead to severe airway rigidity and anatomical distortion, rendering LMA unreliable or even impossible (11, 12).

To address the specific challenge of post-radiation airway distortion, we report a modified single-stage, fluoroscopy-free approach using a thin bronchoscope in parallel with an ETT for airway stenting, achieving safe and effective closure of a post-radiotherapy cervical mTEF with fibrotic airway distortion. This approach leverages the ETT to bypass the distorted upper airway and deliver the stent, the flexibility of the thin bronchoscope for real-time intraprocedural visualization, and continuous mechanical ventilation to minimize procedural risks of hypoxemia and respiratory failure. We also review the relevant medical literature on alternative airway management strategies for cervical mTEF, highlighting the strengths and limitations of our approach and its potential for widespread clinical adoption in both tertiary and resource-limited medical centers.

## 2 Case presentation

The patient was a 79-year-old Han Chinese male, a retired farmer, with a 5-year history of stage IIIB (T3N1M0) thoracic esophageal squamous carcinoma. Five years before the current admission, he had undergone definitive chemoradiation (cisplatin plus paclitaxel chemotherapy combined with 60 Gy radiation therapy), followed by adjuvant immunotherapy (pembrolizumab) for 3 years, achieving a complete response with no evidence of disease progression at regular follow-up. He had no other chronic comorbidities or history of alcohol consumption, with a stable psychosocial status and a supportive family. He had a 20-year smoking history (3 cigarettes daily) with 20 years of smoking cessation at the time of admission.

**Figure 1:**
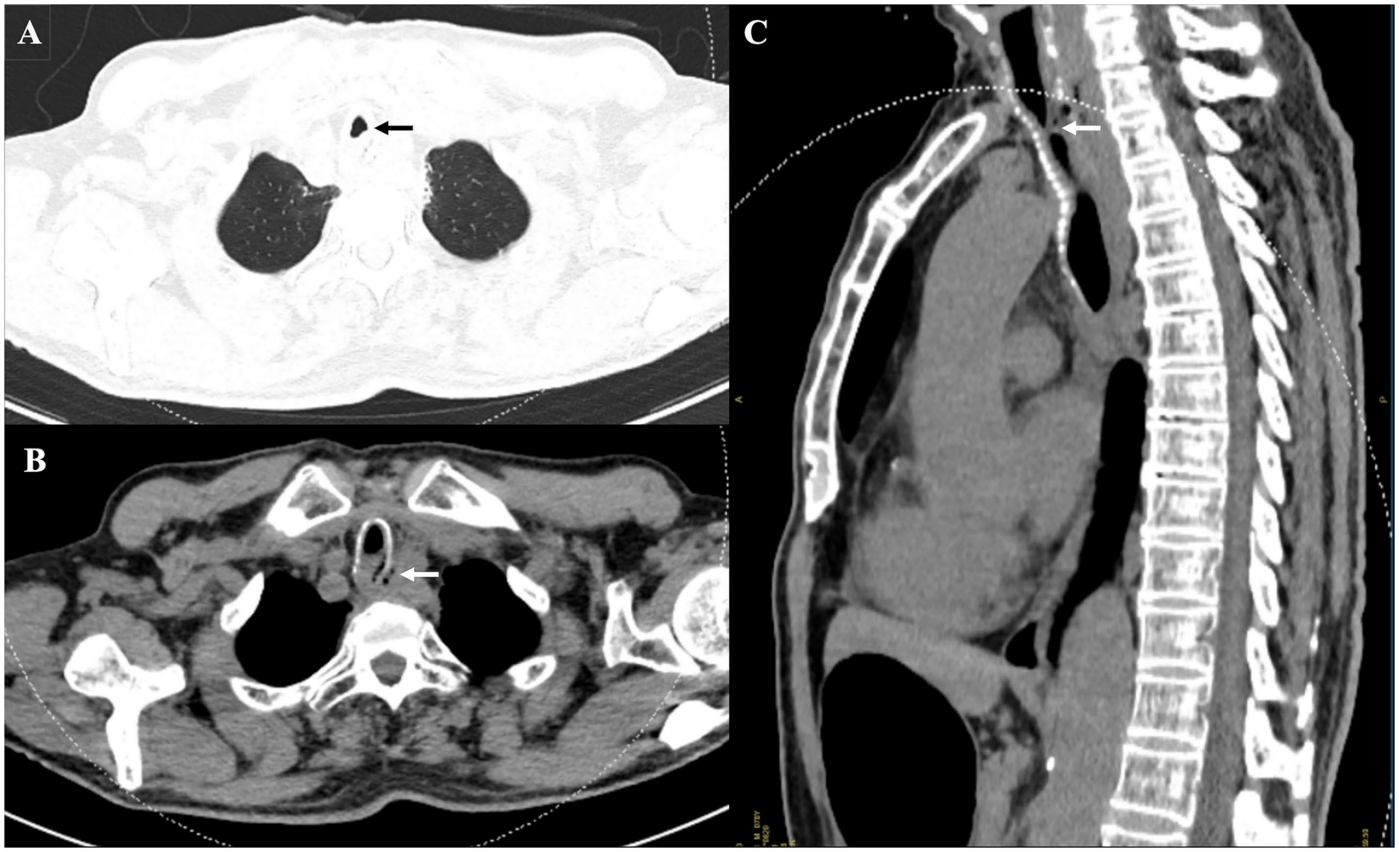
Serial chest computed tomography (CT) imaging at the external hospital. **(A)** Axial CT scan (lung window): moderate cervical tracheal stenosis (black arrow). **(B,C)** Axial and sagittal CT scans (mediastinal window): focal wall thickening of the cervical and upper thoracic esophagus (~1.5 cm), with obliteration of periesophageal fat planes, indistinct tracheal borders, and moderate cervical tracheal stenosis (white arrow). The lesion invades the tracheal lumen with a focal tracheoesophageal fistula, demonstrating the air-containing cavity (white arrowhead).

The patient presented with a 20-day progression of immediate cough on swallowing solids or liquids (Ono’s sign), purulent yellow sputum (30 mL/day), and exertional dyspnea (New York Heart Association Class II). Contrast-enhanced chest computed tomography (CT) from an external hospital demonstrated circumferential thickening of the cervical and upper-thoracic esophagus (indistinguishable from the trachea), intraluminal tracheal involvement, and an air-fluid level consistent with TEF (Figure 1). A video swallow evaluation found significant contrast extravasation from the upper esophagus into the airway. Upper gastrointestinal endoscopy identified an impassable esophageal obstruction at 17 cm from the incisors, and a jejunal feeding tube was placed for nutritional support. Histopathological examination of esophageal tissue revealed high-grade squamous dysplasia with atypical keratinization and malignant transformation (carcinoma); the presence and depth of tissue invasion could not be definitively determined. Flexible bronchoscopy confirmed a small mTEF 5 cm below the vocal cords, accompanied by necrotic tissue but no viable tumor. A diagnosis of mTEF was established; however, stent deployment was deemed unfeasible at the external hospital due to the lesion’s high anatomical location and concurrent distorted upper airway. The patient subsequently developed fever and worsening exertional dyspnea, prompting his transfer to our hospital for further definitive management.

His vital signs were abnormal upon his admission to our department: an elevated temperature of 38 °C, a heart rate of 106 beats per minute, a respiratory rate of 23 breaths per minute, a blood pressure of 122/78 mmHg, and an oxygen saturation of 95% on room air. Bilateral abrasive breath sounds and bilateral moist rales in the lower lobes were identified during physical examination. No other noteworthy findings. The blood routine revealed an elevated neutrophil percentage of 94%, indicative of bacterial infection. Empiric anti-infective therapy with meropenem (1 g per 8 h) targeting Gram-negative bacteria and enteral nutritional support was promptly initiated, and the patient’s fever resolved within 24 h of treatment initiation.

**Figure 2:**
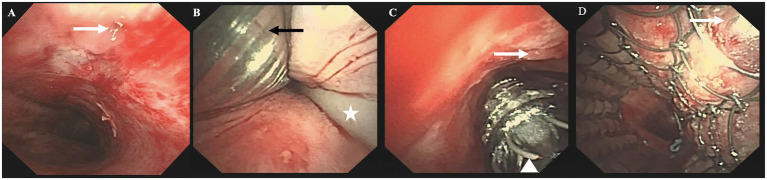
Management of the cervical malignant tracheoesophageal fistula. **(A)** Endoscopic view of a small fistula (white arrow) located 5 cm below the glottis on the left lateral wall of the trachea, with adjacent necrotic tissue. **(B)** Following a 7.5-mm endotracheal tube (ETT) (black arrow) inserted orally with its tip above the fistula, a 3.8-mm thin bronchoscope was passed parallel to the ETT, as demonstrated in the schematic diagram; an existing gastro-jejunostomy tube was indicated (asterisk). **(C)** An 18-Fr stent pusher with a partially covered metallic stent (white triangle) was introduced through the ETT, positioned to cover the fistula (white arrow) under direct bronchoscopic visualization. **(D)** Post-deployment bronchoscopy confirming immediate, airtight closure of the fistula (white arrow).

**Figure 3:**
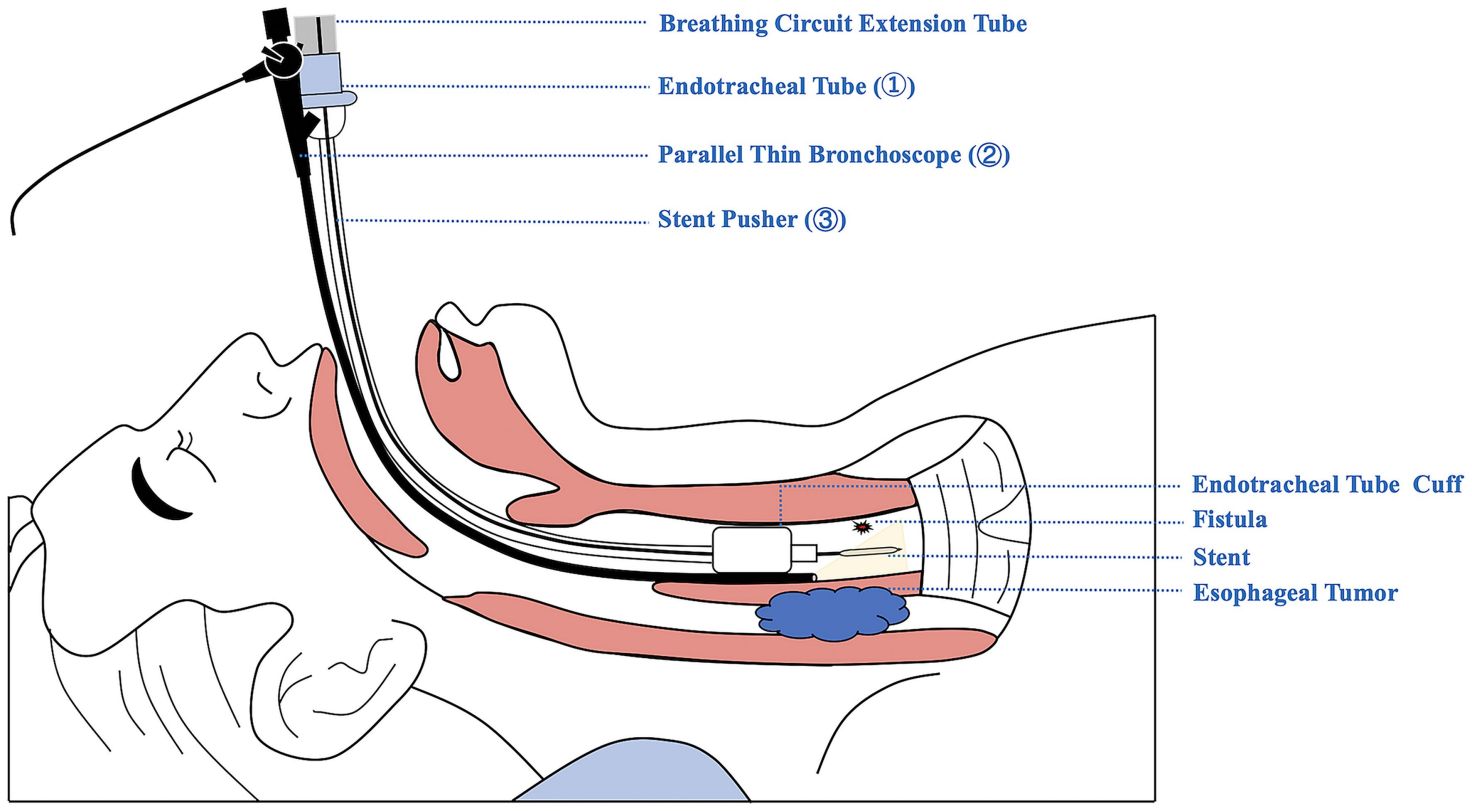
Schematic diagram of bronchoscope-parallel, endotracheal tube-guided stenting for cervical tracheoesophageal fistula. ① The endotracheal tube (ETT) is inserted orally under flexible bronchoscopy, with its tip above the fistula. ② The flexible bronchoscope is inserted orally into the trachea, parallel to the ETT. The cuff is deflated to permit passage and then reflated for mechanical ventilation. ③ The stent pusher is advanced into the trachea via the breathing circuit extension tube to the fistula and deployed under direct bronchoscopic visualization to close the fistula.

Repeat flexible bronchoscopy at our institution confirmed extrinsic compression stenosis of the upper trachea and the presence of a small mTEF on the left lateral tracheal wall, 5 cm below the glottis, with associated necrotic tissue (Figure 2A) and leakage of digestive fluid and air bubbles from the fistula (Supplementary Video S1). The distorted and stenotic pharyngolaryngeal axis precluded a reliable LMA, prompting a direct interventional approach via the tracheal lumen, as demonstrated in the schematic diagram (Figure 3). Under general anesthesia, a 7.5-mm cuffed ETT was advanced orally into the trachea under direct bronchoscopic guidance, with the tip positioned about 1 cm above the fistula by real-time measurement. A 3.8-mm therapeutic thin bronchoscope was inserted parallel to the ETT (Figure 2B) to maintain continuous, real-time intraprocedural visualization. The ETT cuff was transiently deflated during parallel scope insertion, then reflated to ensure a seal for mechanical ventilation. Anesthesia was maintained with continuous infusion of propofol and remifentanil; volume-controlled ventilation was applied with tidal volume 6 mL/kg, respiratory rate 16 breaths/min, fraction of inspired oxygen 50–60%, and continuous monitoring of oxygen saturation and end-tidal carbon dioxide. An 18-Fr delivery catheter loaded with a 20 mm (diameter) × 40 mm (length) partially covered Ultraflex metallic stent (Boston Scientific) was passed through the ETT, positioned to fully cover the fistula under bronchoscopic guidance (Figure 2C), and deployed slowly to prevent misdeployment. Immediate post-deployment bronchoscopy confirmed complete, airtight mTEF closure with no fluoroscopic assistance (Figure 2D). The total procedure time was 45 min, with oxygen saturation maintained at ≥95% throughout via continuous mechanical ventilation.

**Figure 4:**
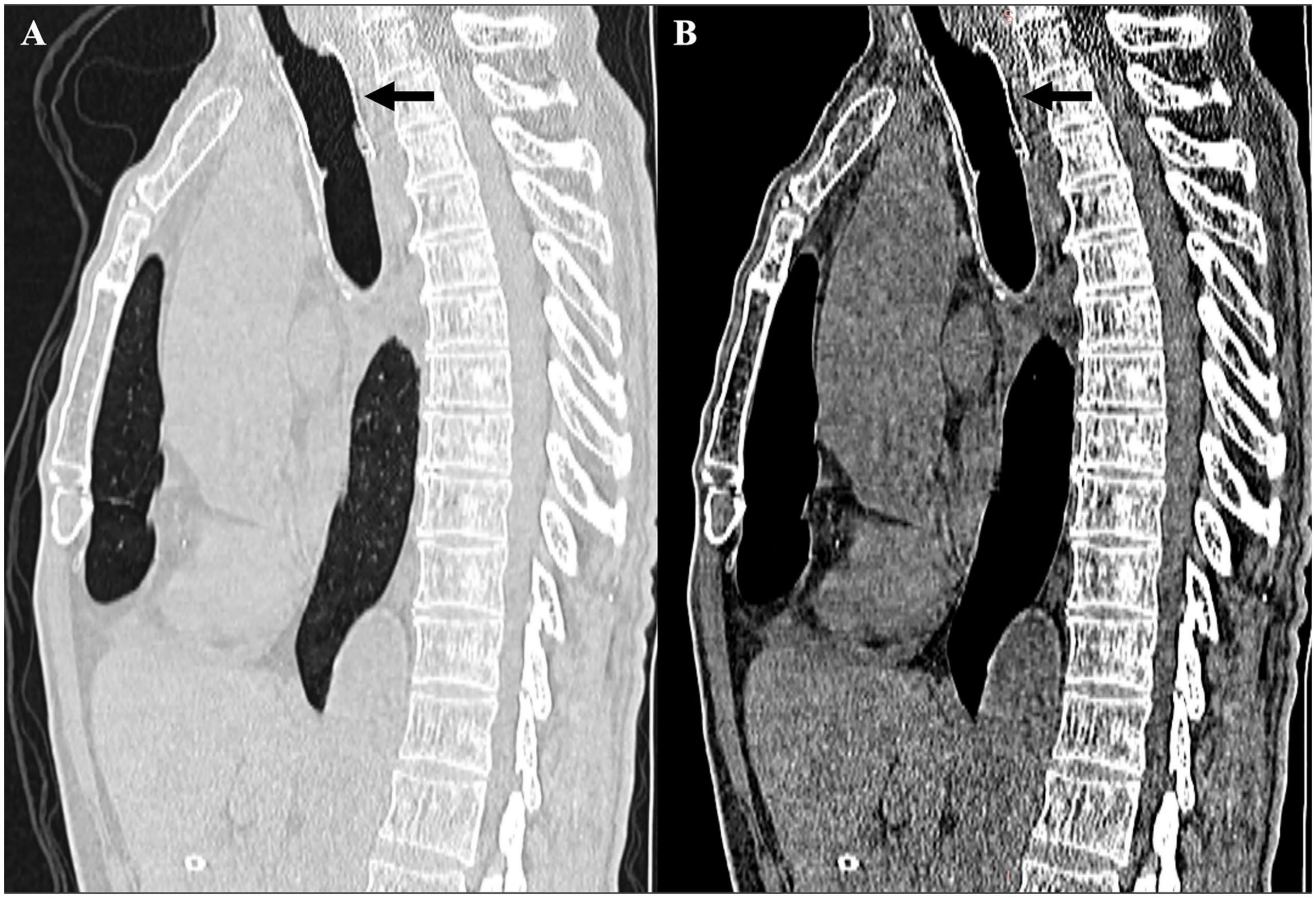
Serial chest computed tomography (CT) imaging after the procedure. **(A,B)** Sagittal CT scans (lung and mediastinal windows): stent shadow seen in cervical trachea (black arrow), with obliteration of periesophageal fat planes. Tracheal patency was improved compared with the previous imaging. The previously noted air-containing cavity had disappeared (black arrowhead), indicating no definite tracheoesophageal fistula.

The patient was extubated immediately following the procedure, initiated on oral clear liquids within 24 h, and transitioned to a complete oral diet by the second day post-stent deployment. The patient reported severe clinical distress before the intervention, describing the swallowing-induced cough as “debilitating” and claiming that it had caused social isolation and unintentional weight loss. In his opinion, the procedure was “painless and efficient,” and the rapid clinical recovery allowed him to resume normal meals with family. The stent was found to be positioned appropriately in the cervical trachea during a follow-up CT scan (Figure 4) 2 weeks post-procedure. The surrounding mediastinal fat was mildly hazy, and no definitive radiological evidence of mTEF was observed. Despite this initial favorable response, the patient developed recurrent coughing and choking on drinking water at 3 months post-procedure and ultimately died after declining further medical and interventional treatment. This short follow-up and limited outcome preclude any conclusion regarding long-term durability; the observed benefit was limited to short-term palliation and immediate symptom relief.

## 3 Discussion

mTEF is a rare but devastating complication of advanced esophageal carcinoma or lung cancer, with a particularly poor prognosis when occurring as a sequela to definitive chemoradiation and immunotherapy (9, 13, 14). In the present case, a 79-year-old patient developed cervical mTEF 5 years after multimodal treatment for stage IIIB esophageal squamous cell carcinoma, representing a delayed and clinically challenging presentation. The formation of such a late-onset fistula is likely multifactorial, involving persistent local tumor recurrence, anti-cancer therapy-induced tissue necrosis, and progressive mediastinal fibrosis, all of which can erode the intervening tracheoesophageal wall despite initial complete response to anti-cancer treatment (15). Clinically, the patient presented with the classic immediate cough upon swallowing (Ono’s sign), recurrent aspiration pneumonia, and exertional dyspnea—hallmark symptoms that severely impair QoL and nutritional status (3). Contrast-enhanced CT and endoscopy remain the cornerstones of diagnosis and management for mTEF, with endoscopy being essential for direct visualization of lesion location, fistula size, associated complications, and treatment allocation (5, 16–18).

Securing the airway during stent deployment represents a core challenge in interventional practice, especially for patients with anatomical distortion or tracheal stenosis (19, 20), where rigid bronchoscopy or LMA was unreliable, necessitating a direct trans-tracheal approach under bronchoscopic guidance in this case. We employed a strategy of thin bronchoscope-parallel, ETT-guided stent deployment, which seems to offer three key advantages: (1) the stiff ETT circumvents the fibrotic and distorted cervical airway and serves as the lumen for stent delivery; (2) the parallel bronchoscope guarantees accurate stent deployment without the need for fluoroscopy; and (3) continuous ventilation mitigates the risk of peri-procedural desaturation, a recognized complication for cervical TEF. This approach, without specified equipment, might be reproducible in medical centers equipped with a thin bronchoscope and a standard anesthesia machine, making it a viable option for resource-limited settings.

Several alternative airway-securing approaches for cervical TEF have been reported in the literature, each with distinct advantages and limitations. A tubeless ventilation approach, supraglottic jet oxygenation through a nasopharyngeal airway, has been demonstrated to maintain effective respiratory parameters during airway stenting (21). This approach eliminates the interference of ETTs or LMAs with bronchoscopic manipulation and stent deployment, creating sufficient space for complex stent insertion. However, it is not recommended for patients with extensive oropharyngeal edema, glottic malformation, or an impaired cough reflex, as it is contingent upon optimal glottic closure and upper airway patency (22). Additionally, improper jet pressure regulation may cause pulmonary barotrauma, and the lack of a sealed airway increases the risk of aspiration and carbon dioxide retention, especially in patients with poor protective airway reflexes; it also requires proficient operational skills, raising the threshold for clinical application (23). Another viable approach is the utilization of a double-lumen ETT, which has two independent lumens (24). The monitoring lumen facilitates the passage of a thin, flexible bronchoscope, allowing for real-time visualization of the insertion process, while the operative lumen functions as a pathway for stent delivery, facilitating precise deployment and release. This technique shares the same fundamental principle as our approach—a procedure conducted under general anesthesia with bronchoscopic guidance to ensure interference-free stent deployment. However, it is constrained by reliance on a specialized, custom-fabricated tube. The non-availability of this bespoke tube, its associated costs, and preparation time significantly hinder its widespread clinical adoption, especially in resource-limited institutions. Our ETT-parallel thin bronchoscope approach, in contrast, circumvents these constraints and offers a promising alternative for post-radiation distorted and stenotic airways where LMA placement is impractical.

Stent fixation represents another critical consideration, particularly for cervical (upper tracheal) stents. Unlike gastrointestinal stents, which can be adjusted and secured using transoral threads or sutures (25), upper tracheal stents pose unique challenges that render such fixation methods impractical. Alternative fixation strategies have been investigated, such as stents with specialized design features (e.g., hourglass-shaped silicone stents with flared ends or circumferential barbs) to enhance anchorage (20). However, the efficacy of these design features is diminished in post-radiotherapy airways with fibrotic, rigid tissue, as the tracheal wall is unable to deform sufficiently to accommodate barbs or flared ends. In this case, a partially covered stent with a diameter of 20 mm was used; the covered portion completely closed the fistula, while granulation tissue formed at the uncovered portion to reduce stent migration. Meanwhile, the larger diameter (2 mm larger than the normal tracheal diameter measured by CT) also played a role in reducing stent migration to a certain extent. Ensuring optimal stent size—with a diameter slightly larger than the tracheal lumen to exert adequate radial force—is critical for stable anchorage. External fixation is another reported method, demonstrating efficacy in mitigating stent migration (a weighted mean migration rate of 3.3% with few or no reported complications) (26, 27).

A multidisciplinary strategy incorporating interventional pulmonology, anesthesiology, and radiology is essential for high-risk cases, such as post-radiation patients with anatomical distortion (20). The selection and deployment strategy of stents is guided by pre-procedural imaging, which involves contrast-enhanced CT or bronchoscopy, evaluating tracheal anatomy, lesion location, and tissue quality. Patient education to avoid activities that increase intratracheal pressure (e.g., excessive coughing, straining) is equally important as post-procedural care, which includes close monitoring of symptoms, regular bronchoscopic follow-up, and stent migration prevention.

This study has several important limitations. First, as a single-case report, the findings lack statistical power and broad generalizability; the ETT-parallel thin bronchoscope approach cannot be confirmed as widely applicable to all patients with post-chemoradiation late-onset mTEF, and only preliminary feasibility is demonstrated. Second, the 3-month short follow-up restricts assessment of long-term outcomes, including fistula recurrence, stent migration, granulation tissue hyperplasia, and procedural durability, with no evidence supporting long-term efficacy. Third, no controlled comparison with conventional airway management strategies (e.g., rigid bronchoscopy, LMA-guided stenting, double-lumen ETT technique) was conducted, so no definitive conclusion can be drawn on the superiority or equivalence of this method. Fourth, while key procedural details were added, full reproducibility still requires multi-center validation. Fifth, the patient’s death at 3 months post-procedure limits outcome interpretation, and long-term survival or durable palliation cannot be evaluated. Finally, the reference list is over-reliant on case reports, which reduces the strength of evidence for contextualizing and validating the approach.

## 4 Conclusion

Late-onset mTEF should be suspected in patients with prior esophageal cancer who present with the new-onset swallowing-related cough. In this single-case experience, the ETT combined with a parallel thin therapeutic bronchoscope showed feasible and short-term safe performance as a fluoroscopy-free strategy for closing high-positioned mTEF in post-radiation airways unfit for LMA placement or rigid bronchoscopy. This approach uses conventional equipment, is easy to operate, and may help reduce procedural risks in patients with anatomical distortion, suggesting it could be a practical option for short-term palliative care in resource-limited medical centers. Individualized airway management, precise stent selection and deployment, and multidisciplinary collaboration remain critical for improving symptom control in this high-risk patient population, although late-onset post-chemoradiation mTEF has an inherently poor long-term prognosis. Given the limitations of a single case and short follow-up, our findings are preliminary and hypothesis-generating; further controlled studies with larger sample sizes and extended observation periods are required to validate the long-term safety, efficacy, and reproducibility of this approach, as well as to compare it with other established airway management strategies for this specific patient group.

## Data Availability

The original contributions presented in the study are included in the article/Supplementary material, further inquiries can be directed to the corresponding author.
